# Vibration Measurement at Outlet Nozzle for Optimizing Steel Grit Blasting

**DOI:** 10.3390/s26165281

**Published:** 2026-08-20

**Authors:** Milan Sigmund, Tomas Fryza, Jiri Schimmel, Jiri Neuwirth

**Affiliations:** 1 Faculty of Electrical Engineering and Communication, Brno University of Technology, Technicka 3082/12, 61600 Brno, Czech Republic; fryza@vut.cz (T.F.); schimmel@vut.cz (J.S.); 2 Faculty of Mining and Geology, Technical University of Ostrava, 17. listopadu 2172/15, 70800 Ostrava, Czech Republic; jiri.neuwirth.st@vsb.cz

**Keywords:** abrasive blasting, vibration, accelerometers, LPC spectrum, signal processing

## Abstract

This paper deals with enhanced control of blasting via processing of vibration signals. In the blasting process, a mixture of abrasive and air under pressure is fed to the workpiece. However, the pressure set by the operator in the compressor is not always the same as the pressure in the end nozzle; it can decrease due to the passage of the mixture through a longer supply hose. Our research was focused on classifying vibration signals measured in the outlet nozzle according to the mixture pressure. The developed algorithm is based on a smooth spectrum of vibrations obtained by the linear prediction method. To achieve optimal results, vibration signals from several sensing options were measured and compared in tests involving three types of accelerometers and three different positions of accelerometer placement on the nozzle, with regard to vibrations in the axial and radial directions. The best classification accuracy of 90.8% was achieved for a pressure resolution of 2 bars when measuring axial vibrations on the nozzle holder. Monitoring the outlet pressure of the mixture can improve existing blasting systems by improving the ability to automatically maintain optimal operating pressure during blasting.

## 1. Introduction

Abrasive blasting is one of the most widely used surface treatment technologies in industry. The purpose of blasting is most often to remove unwanted impurities from the surface of materials before further surface treatments [1] (cleaning, peening, and finishing various materials) or to achieve a suitable roughness on the surface of the processed product. In the blasting process, abrasive particles are impinged under pressure with high kinetic energy on the targeted surface. The efficiency of blasting depends on several factors, the most important of which are listed as follows:

- type of abrasive and particle size;
- diameter and internal shape of nozzle;
- mixing ratio in the abrasive and air mixture;
- pressure of the mixture during blasting;
- distance and angle of blasting;
- type of blasted material.

From a process-control perspective, two parameters are particularly important: air pressure and abrasive flow rate. Air pressure directly affects particle velocity and impact energy. Increasing the pressure generally increases material removal rate and cleaning efficiency, but may also lead to excessive surface roughness, increased abrasive consumption, and accelerated nozzle wear. Maintaining stable pressure is therefore essential for achieving repeatable blasting results.

A typical blasting system is comprised of three basic components: an air compressor, a blast machine, and an abrasive. The compressed air provides the driving force, while the blast machine doses the abrasive into the air stream. Systems are generally categorized as suction (venturi) or pressure types, with pressure systems typically offering much higher particle velocities and productivity.

The nozzle plays a critical role in determining production speed and the final appearance of the surface. Its geometry influences the air velocity, abrasive acceleration, and spray pattern. However, nozzles are subject to rapid wear. When a nozzle orifice enlarges by just 1.6 mm (1/16 inch), air volume requirements can increase by 25%, leading to significant energy waste and a less consistent process [2]. Modern blasting systems increasingly incorporate sensors for pressure, air flow, and abrasive consumption, enabling continuous monitoring and more precise active control in real time using automatically adjusted processing modes [3].

In general, the type of abrasive is chosen according to the properties of the surface to be treated, the degree of contamination, and the thickness of the object walls. Objects made of soft or thin-walled materials are blasted at lower pressure with finer grains, while massive objects can be blasted at higher pressure with coarser grains. Due to their physical and chemical properties, the most frequently used abrasives for surface cleaning and pre-treatment applications are steel grit, steel shot, corundum, and sand. The particles of steel abrasives can be round (i.e., steel shot) or angular (i.e., steel grit). Steel abrasive materials used in industry commonly have a particle size range between 1 mm and 8 mm. The chemical composition of steel grit usually includes 94% iron and trace elements such as calcium oxide, aluminum oxide, manganese oxide, and others. Our study is focused on dry blasting with steel grit.

Blasting can be carried out in an enclosed space or in an open space. Enclosed spaces are designed and equipped specifically for a certain type of blasting and are usually part of a production or service facility. Depending on the size of the object to be blasted, a blasting chamber (for smaller, portable items) or a blasting box (for larger items) can be used. Typically, an entire car can drive into the blasting box. In these cases, the chamber or box is a permanently installed part of the entire blasting equipment, and the set parameters of the blasting process are directly applied when blasting.

Another situation can occur when blasting in an open space. Here, the object to be blasted is fixed in a certain place, it is often not possible to place the blasting source in the immediate vicinity of the blasting object, and the distance between the source and the blasting target must be bridged by a hose for transporting the blasting medium. In such a case, pressure loss occurs in the hose. In addition, the output pressure deviation is not constant at a given workplace, but can fluctuate over time, especially if the trajectory of the hose changes.

Depending on the desired blasting goal, there is an optimal combination of mixture pressure and mixture composition for each blasting process. For example, when restoring vintage vehicles, a smaller amount of abrasive in the mixture and a finer flow of mixture are required for blasting bodywork than when blasting welds on pipes or building structures. The primary effect of correctly tuning the parameters is lower abrasive consumption. Lower financial costs are directly related to the saving of abrasive, and a lower environmental burden. From a technological point of view, a higher quality of surface finish is achieved on the machined object, and the necessary blasting time is shortened. The desired combination of optimal parameters is usually known in advance and is set by the operator on the blasting equipment. However, if the blasting parameters change uncontrollably, it is desirable to automatically adjust them to the desired values.

The aim of the presented study is to show the potential of vibration signals at the nozzle for non-invasive determination of the actual pressure. This study reports research into the optimal use of sensors and the first results of the classification of vibration signals. The rest of this paper is organized as follows. A brief overview of related works is given in Section 2. Section 3 introduces vibration sensors (accelerometers) and their relevant properties. The hardware equipment, signal data, and processing methods are described in Section 4. The experimental results obtained in the tests are presented and discussed in Section 5. Finally, Section 6 concludes this paper.

## 2. Related Works

To our knowledge, no work on measuring vibrations at the outlet nozzle and processing this data exists (or it is not available). However, there are publications dealing with related topics.

Traditional industrial methods for powder blasting control, such as the use of Almen strips, provide general process information but fail to offer the real-time feedback necessary for maintaining optimal functionality [4]. Modern production requirements require continuous tracking and stabilization of variables like jet kinetic power, which is prone to significant fluctuations over time due to pump drifts, unstable abrasive feeding, and progressive equipment wear [5]. Consequently, there is a strong shift toward non-invasive, inline monitoring systems that enrich available datasets and enable closed-loop control to ensure stringent quality and safety standards.

Acoustic emission (AE) sensing involves the detection of transient elastic waves, typically with broadband frequency spectra between 0.1 and 1 MHz, generated by the rapid release of energy from localized sources such as particle impacts, friction, or plastic deformation [6,7,8]. The energy of these stress waves is directly proportional to the incident kinetic energy of the particles, scales with the square of the particle velocity, and exhibits a linear relationship with mass concentration [6,9]. Specific research results indicate that AE sensors attached to fixed meshes within a pipeline can measure average particle velocity with reasonable accuracy using transit-time calculations, while in waterjet cutting applications, the root mean square value of the AE signal serves as a sensitive tool for monitoring penetration depth and material removal rates [7,10].

Electro-acoustic monitoring utilizes microphones to capture airborne acoustic pressure waves generated by the process and convert them into electrical signals [4,11]. Experimental data show that microphones can successfully monitor changes in nozzle-to-substrate standoff distances, detect the edges of substrates during automated scanning, and track nozzle wear by identifying shifts in acoustic longitudinal modes modeled as a quarter-wavelength air column.

Vibration analysis employs accelerometers to measure the mechanical oscillations generated by the impact of abrasive particles on containment walls or target surfaces, releasing a fraction of their kinetic energy as vibration waves [12,13]. In gas and sand conveyor systems, vibration sensors can effectively detect trace amounts of particles as small as 45 μm, with measured vibration energy correlating to sand mass flow rate with uncertainties below ±8% [13]. Furthermore, the use of statistical indicators such as generalized norms and moments extracted from vibration signal envelopes has proven highly accurate for estimating powder mass flow rates in mechanical screw feeders, as these higher-order norms are particularly sensitive to the impact bursts of moving particles [8].

## 3. Vibration Sensors

In this study, vibration measurements were performed using piezoelectric shear-type accelerometers. The design of these sensors is based on piezoelectric plates placed between a central post and a seismic mass [14]. A preload force is applied to the assembly by means of a compression ring or stud, which ensures high structural rigidity and linear response of the sensor. When accelerated, the seismic mass induces shear stress in the piezoelectric elements. Due to the piezoelectric effect, this mechanical stress generates an electrical signal proportional to the applied acceleration. The ThetaShear accelerometer employs two parallel piezoelectric plates with a seismic mass placed between them. The resulting electrical signal is collected by electrodes and transmitted by lightweight lead wires either to the sensor’s integrated electronics for signal conditioning or directly to the electrical connector in charge-mode accelerometers [15]. The design principle of shear piezoelectric accelerometers is depicted, for example, on the website [16].

Three different types of accelerometers (hereinafter referred to as A1, A2, and A3) with different dynamic and frequency ranges were tested. Photos and details of the sensors can be found in the datasheets [17,18,19] and their basic parameters are listed in Table 1. Type 4517 excels in frequency range, but its inherent noise is more than ten times higher that of the other two accelerometers. Furthermore, the upper frequency limit is significantly affected by adhesive mounting, and calibration would be necessary to determine it accurately. The adhesive mounting method introduces a flexible intermediate layer at the sensor–structure interface, leading to damping of high-frequency vibrations and a corresponding reduction in the resonant frequency of the assembly. The 4534 and 4507 accelerometers have the same sensitivity and very similar noise; the 4534 type has a higher dynamic and frequency range. However, its disadvantage is a much higher price. All three types have a transverse sensitivity less than 5%. During the verification measurements, it was verified that the dynamic range and frequency range of the 4507 accelerometer were sufficient.

## 4. Materials and Methods

This section presents relevant information about the conditions under which the experimental results were obtained.

### 4.1. Measuring Hardware and Instrumentation

**Figure 1:**
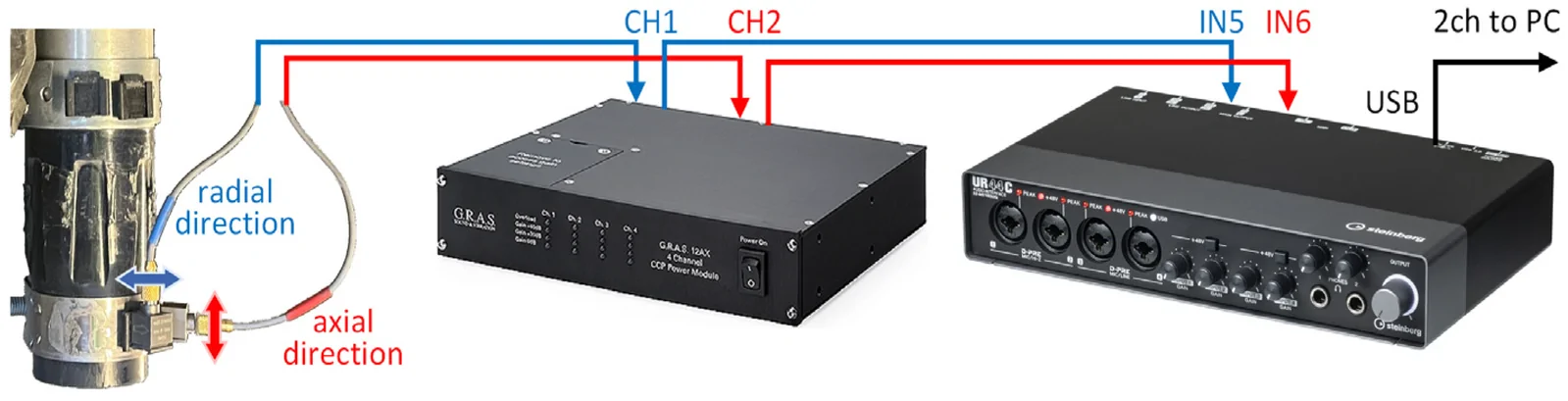
Diagram of the measurement circuit with radial (–) and axial (–) vibration signals.

All accelerometers used are of the Constant Current Line Drive type. A 4-channel CCP Power Module GRAS 12AX [20] (GRAS Sound & Vibration, Holte, Denmark) with adjustable gain at 0, 20, and 40 dB and a maximum gain error of ±0.15 dB was used as a preamplifier. This allowed compensation for the voltage sensitivity of the A2 accelerometer, which was approximately 20 dB lower than the sensitivity of the other two. The output voltage was recorded into a personal computer using a Steinberg UR44C [21] audio interface with a sampling rate of 48 kS/s and a resolution of 16 bits. The connection of the measuring station is shown in Figure 1.

According to the parameters of the accelerometers listed in Table 1, the residual broadband noise voltage of accelerometer A1 was equal to 50 µV, A2 was equal to 60 µV, and A3 was less than 34 µV. The theoretical output voltage corresponding to the acceleration measurement range was approximately 7 V for accelerometers A1 and A3 and 5 V for accelerometer A2. Since the lower and upper limits of the dynamic range of the three accelerometers are close to each other, we consider the sensor dynamic range to be approximately 0.05 mV to 5 V, i.e., 100 dB. The GRAS preamplifier provided a sensor supply voltage of 28 V and its inherent noise for the 20 dB gain used for the A2 accelerometer was 15 µV. The frequency response (+1, −3 dB) for this gain is 1 Hz to 200 kHz. The preamplifier did not cause any limitation of the dynamic or frequency range of the accelerometers.

The audio interface used had a maximum input voltage of 9.7 V and an inherent noise of 78 µV (A-weighted). Since the minimum output voltage of an accelerometer (when no blasting is taking place) is affected by the idle vibrations of the device, the dynamic range of the audio interface is sufficient. However, limitation of the dynamic range may be caused by the quantization noise, the theoretical value of which is 0.3 mV for the bit resolution used. If we require the signal-to-noise ratio of 20 dB at minimum measurable vibration, the minimum vibration should be 30 mg (assuming a 20 dB gain for the A2 accelerometer). During verification measurements, it was found that this condition was met.

The audio interface manufacturer states only a 0.4 dB drop in frequency response at 20 Hz. To compare the frequency range with that of the preamplifier integrated in the GRAS 12AX Power Module [20], we performed a measurement that showed a 3 dB drop at frequency of 2.5 Hz. During preliminary analysis, it turned out that this limitation of the measurement frequency range was not a problem, because the vibrations caused by blasting did not exhibit any significant energy in this part of the spectrum.

### 4.2. Data Measured

There is no available database containing records of vibration signals generated in the outlet nozzle during blasting. Therefore, we employed only signals from our own database created as part of the research project stated in the *Funding* paragraph.

The recordings were made for 4 types of steel grit abrasive GH120, GH80, GH40, and GH18 (Steel Works TAS, Kamianske, Ukraine), two nozzles with different inner diameters (6.4 mm and 8.0 mm), and different combinations of pressure and mixing ratio in the abrasive and air mixture during blasting. The pressure varied stepwise from 3 bar to 7.5 bar in increments of 1 or 0.5 bar. The fine pressure increase of 0.5 bar was used only for the values 5.5, 6.5 and 7.5 bar. The range from 5.5 to 7.5 bar covers the optimum pressure values for blasting in most situations. The mixing ratio was varied from 0% abrasive (i.e., only compressed air was blasted) to 100% abrasive in 10% or 30% increments. The blasting parameters did not change during the recording of each individual signal. The duration of each recording was between 30 and 405 s. Most vibrations were measured in pairs, synchronously in the radial and axial directions. The vibration signals were sampled with a sampling rate of 48 kS/s, 16 bits, mono and saved in wav format. In total, the database used contained 784 vibration signals under various conditions with a data volume of 2.9 GB. However, not all complete data sets of pressure and mixing ratio combinations were measured for all steel grits and both nozzles.

**Figure 2:**
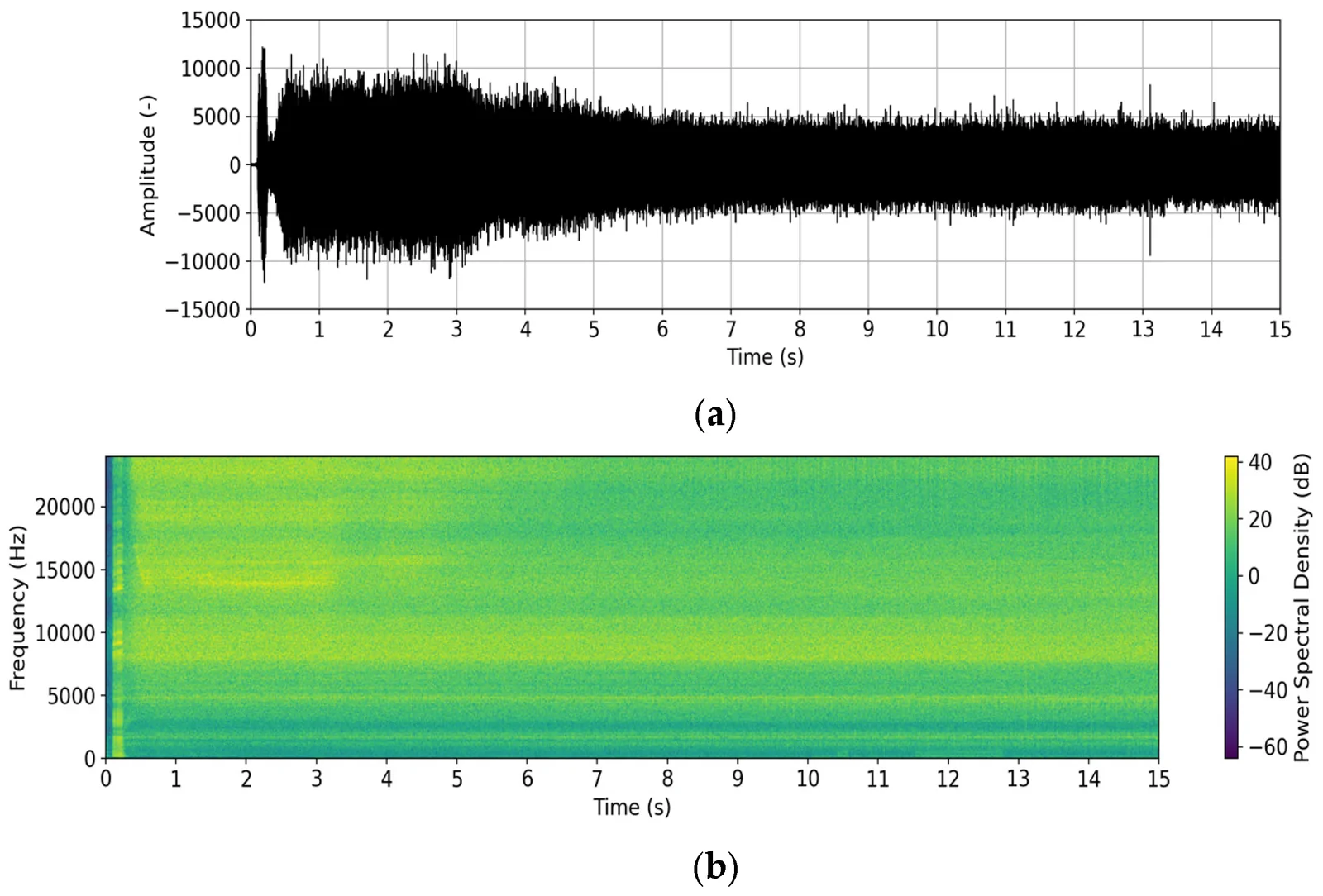
Vibration signal (**a**) and its corresponding spectrogram (**b**) when blasting with 60% steel abrasive, pressure 6 bar, and nozzle 6.4 mm.

**Figure 3:**
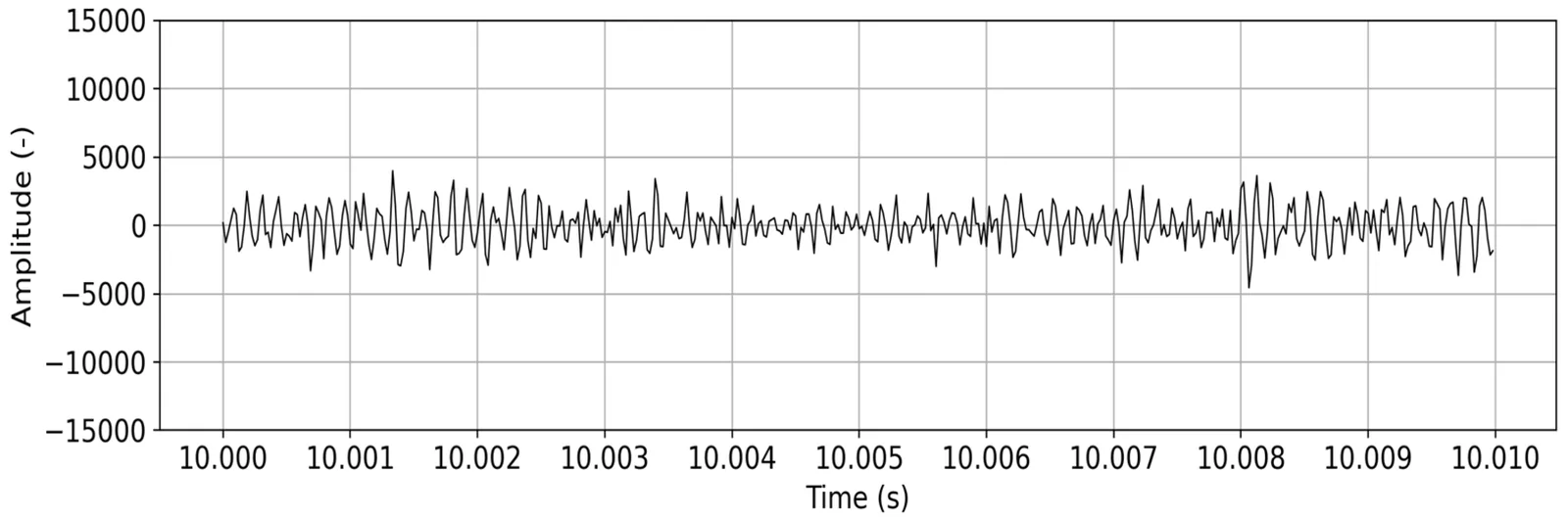
Details of short steady vibration extracted from the signal in Figure 2a.

In general, the measured vibration signals had a noisy character. Figure 2 illustrates the raw signal of axial vibration acquired by the A3 accelerometer and recorded at a sampling rate of 48 kS/s along with the corresponding spectrogram. Essentially, the diagram shows the signal envelope due to the high density of the recorded data. However, the initial start-up phase, during which the first abrasive particles travelled to the nozzle (3.5 s), as well as the moment when the abrasive flow reached a steady state (6 s), is clearly recognizable. Figure 3 shows a detailed 10 ms waveform cut from the signal in Figure 2 in the time span from 10.000 to 10.010 s, which was within the steady-state blasting. Vibration processing in all algorithms presented below was performed with steady signals.

### 4.3. Smooth Spectrum Based on Linear Prediction

To capture the spectral trend well, we used smooth spectra derived from linear prediction in our experiments. Determining the signal spectrum using linear prediction can be considered an alternative to the Fourier transform. Linear prediction was originally defined for speech coding applications and quickly became used in many other areas of signal processing. The term “linear predictive coding” or abbreviated as “LPC” is now often used as a term for linear predictive analysis in general, i.e., not limited to “coding”. LPC analysis is well established in audio signal processing, see for example [22,23]. In speech research, methods based on linear prediction can be apply for special analyses, such as emotion and stress recognition [24,25], distinguishing gender by voice [26], Parkinson’s disease detection [27], etc.

The basic concept of linear predictive analysis is to predict the *n*-th sample of a signal by means of a linear combination of its previous weighted samples.

(1) $$\hat{s}\left(n\right)=\sum_{m=1}^{M}a_{m}s\left(n-m\right)$$

where *a_m_* are the prediction coefficients (also called LPC coefficients) and *M* denotes the number of LPC coefficients used in the predictive model (i.e., the order of the predictor). Equation (1) can be rewritten in *z*-transform notation as follows:

(2) $$\hat{S}\left(z\right)=\sum_{m=1}^{M}a_{m}z^{-m}S\left(z\right)$$

which leads to a normalized transfer function of the predictor with unit gain, as follows:

(3) $$H\left(z\right)=\frac{1}{1-\sum_{m=1}^{M}a_{m}z^{-m}}.$$

Denoting the sampling frequency of a discrete signal by *f*_sam_, and setting *z* = exp(j2π*f*/*f*_sam_), the magnitude spectrum of the signal can be estimated as follows:

(4) $$S\left(f\right)={\left|\frac{1}{1-\sum_{m}a_{m}z^{-m}}\right|}^{2}=\frac{1}{{\left|1-\left(a_{1}e^{-j2\pi f/f_{\mathrm{sam}}}+a_{2}e^{-j4\pi f/f_{\mathrm{sam}}}+\ldots +a_{M}e^{-j2M\pi f/f_{\mathrm{sam}}}\right)\right|}^{2}}.$$

**Figure 4:**
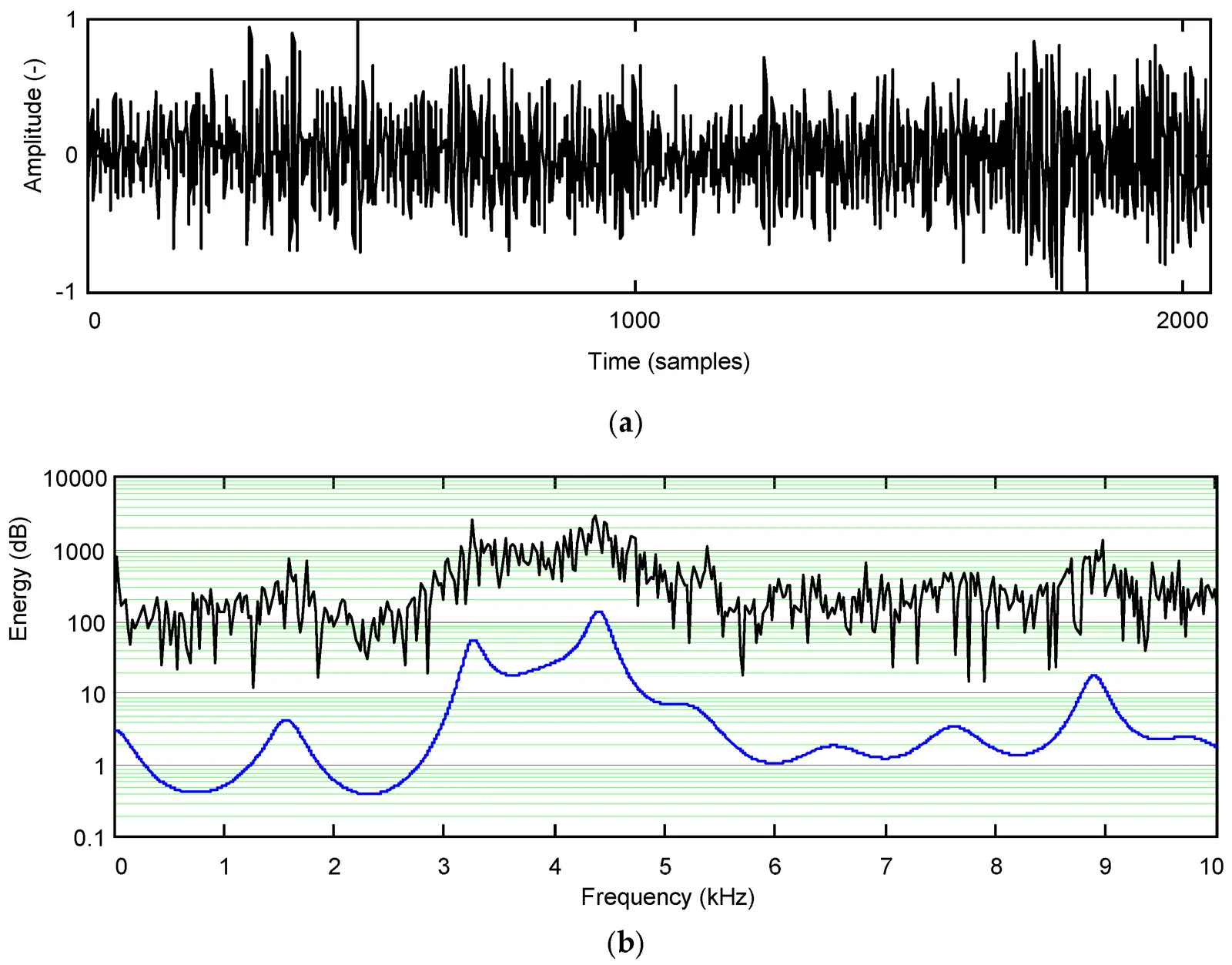
Short-time input signal (**a**) and comparison of spectra obtained by FFT and LPC methods (**b**). In graph (**b**), the FFT spectrum (upper black line) is shifted relative to the LPC spectrum (lower blue line) for better graphical clarity.

To show the nature of the LPC spectrum, Figure 4 graphically compares log spectra obtained by both fast Fourier transform (FFT spectrum) and linear prediction (LPC-spectrum). As can be seen, the linear predictive spectrum represents the general shape and is much smoother than the FFT spectrum.

**Figure 5:**
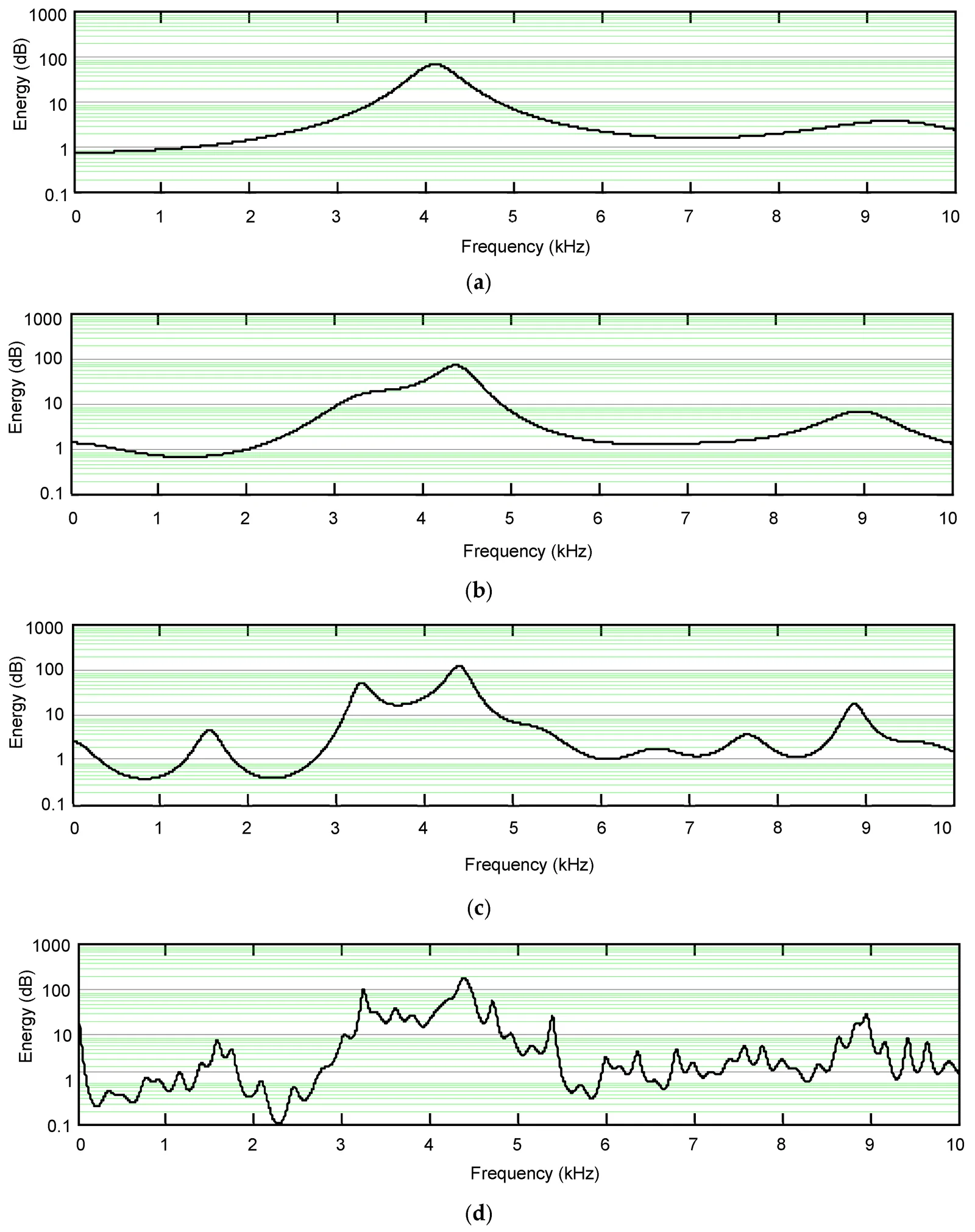
Effect of the prediction order *M* on the resulting LPC spectrum of a signal sampled at 48 kS/s illustrated for several values: *M* = 10 (**a**), *M* = 20 (**b**), *M* = 50 (**c**), and *M* = 250 (**d**).

One of the significant properties of the LPC spectrum is that the order *M* of the linear prediction can effectively control the degree of smoothness of the obtained spectrum. Figure 5 shows the effect of increasing *M*. As *M* increases, the spectrum contains more detail. This is a rule of thumb, but the degree of smoothing is also determined by the sampling rate. The higher the sampling rate, the higher order *M* is necessary to display more detail. The optimal value of *M* should be found according to the optimal shape of the spectrum in each application. In our experiments, the order of *M* = 20 was used.

In the above linear prediction theory, it is assumed that the LPC coefficients are known. Computation of LPC coefficients can be easily carried out in programming languages such as MATLAB or Python using built-in functions. For a better insight into the computation of LPC coefficients, the entire algorithm is described in detail in Appendix A.

A comprehensive overview of linear predictive analysis and derived techniques can be found, for example, in [28,29].

## 5. Experimental Results and Discussion

The experiments were conducted to find the optimal application of accelerometers and processing of captured vibration signals with the aim of estimating the current blasting pressure at the blasting point. The abrasive used in the tests was steel grit.

### 5.1. Examining the Stationarity of Vibration Signals

**Figure 6:**
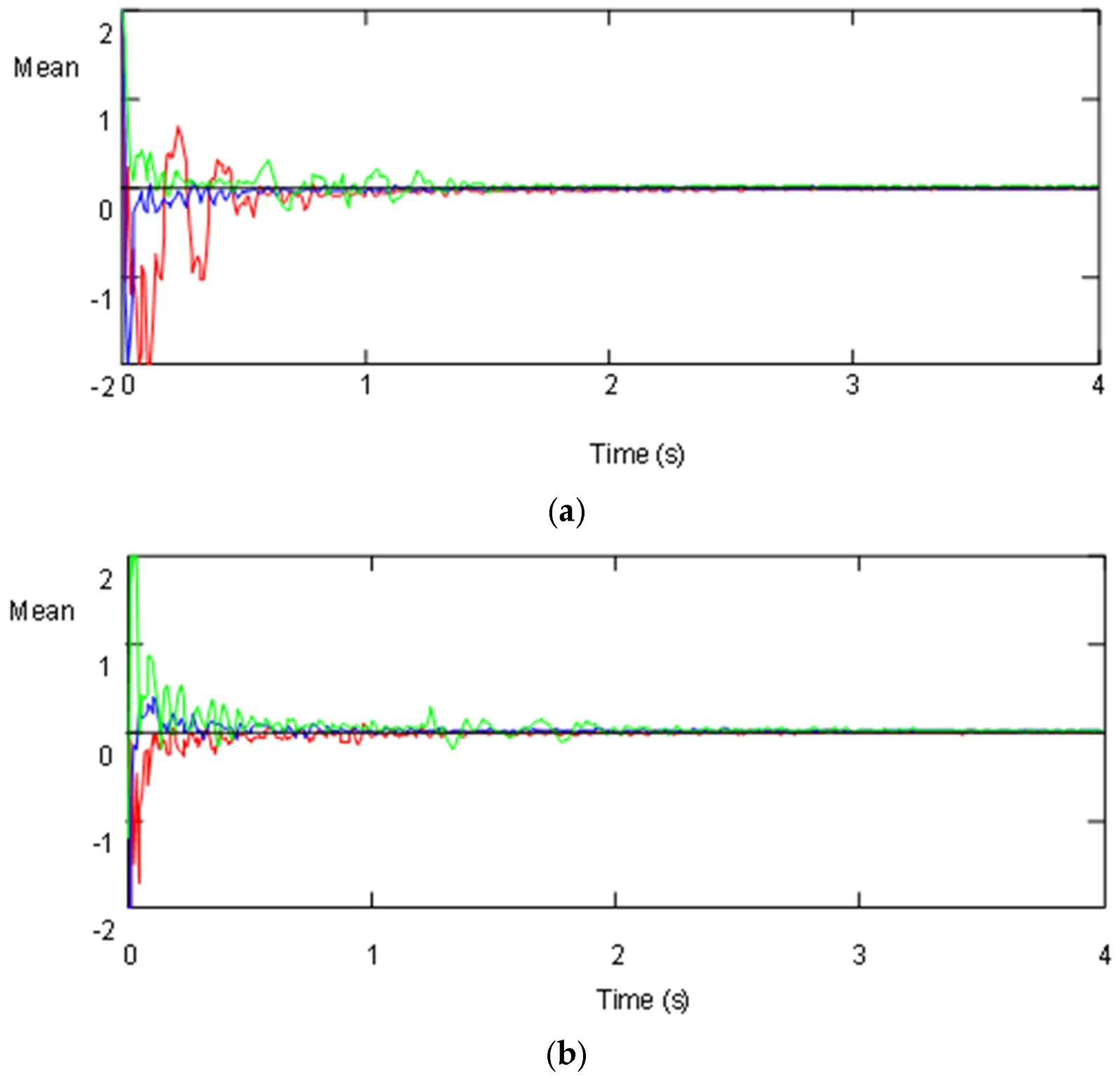
Plot of cumulative mean values for pressure of 3 bar (**a**) and 7 bar (**b**) with 50% abrasive.

The physical nature of the process that generated the vibrations to be analyzed suggested that the vibration signals could have been stationary. Initial experiments were aimed at validating this assumption and finding the necessary length of signal segments to satisfy the stationary behavior. Stationarity represents the ability of a given signal to exhibit the same properties in different parts of its waveform. In practice, this means that we can take a short segment from the signal at any time and determine the properties of the entire signal from it. The necessary length of the segment taken is important. Stationarity was simply examined in the time and frequency domains. Figure 6 shows the development of the signal mean in time analysis of radial vibrations from the A3 accelerometer.

In each graph in Figure 6, three curves estimated from different segments starting at randomly selected times are plotted. In general, the values were more dynamic at lower pressure, but they did not change from 2 s in any of the tests.

**Figure 7:**
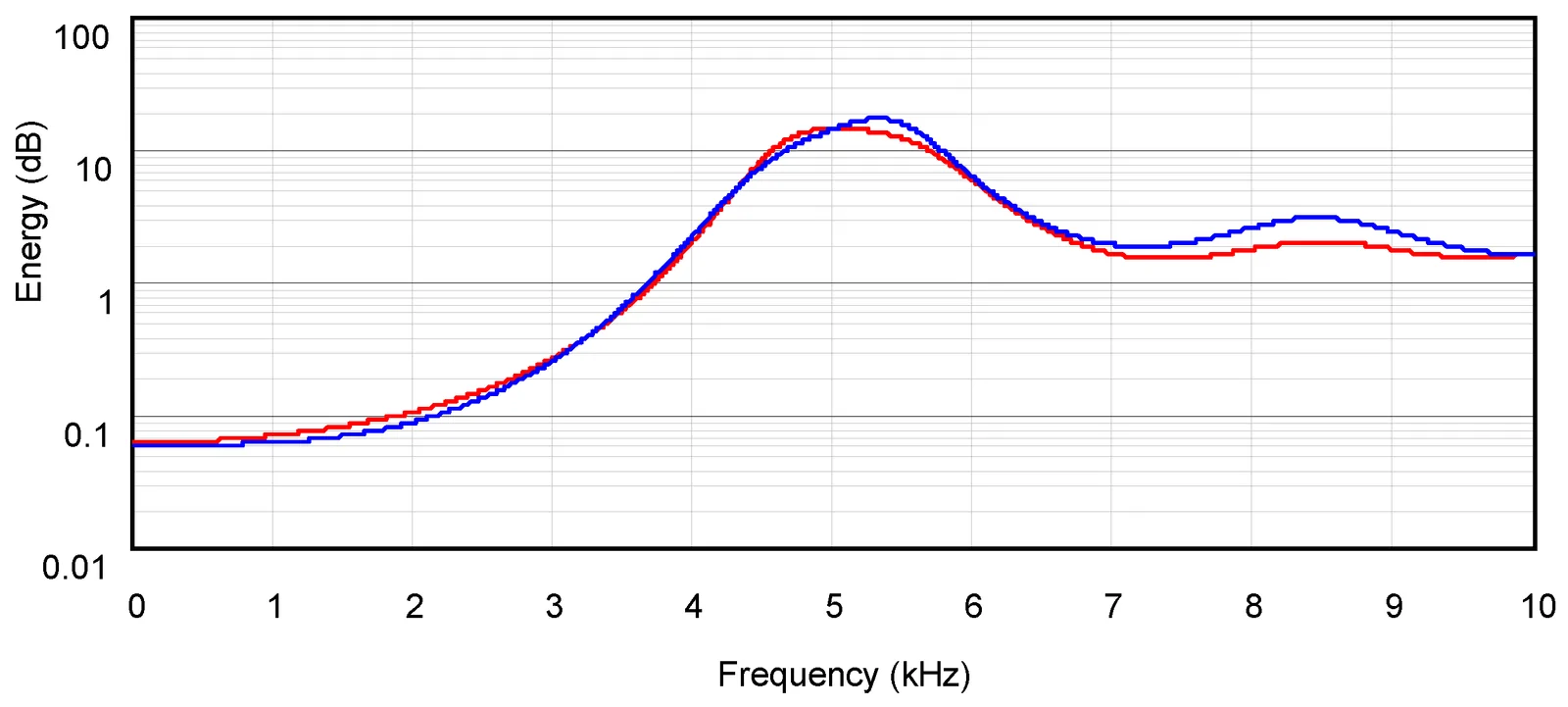
Spectrum variability for 5-s segments at a pressure of 7 bar.

To prove stationarity in the frequency domain, the variability of smooth spectra was examined as a function of increasing signal length. The spectra of two equally long segments extracted from different parts of the signal were compared. As the length of the segments increases, the differences in the spectra become smaller. Ideally, the spectra from all parts of the signal should be identical. Figure 7 illustrates the radial vibration spectra at 7 bar pressure and 50% steel abrasive from the A3 accelerometer. Comparing the times to reach steady state for the waveform mean and the variability of smooth spectra, the spectra required approximately twice as much time. Based on tests performed with signals generated at different pressures, abrasive ratios, and in two vibration directions, it can be concluded that vibration signals behaved as stationary when a segment of 5 s or longer was processed.

### 5.2. Continuous Detection of Vibration Abnormalities

There may be some abnormalities in the captured vibration signals generated by various sources. The most common abnormalities are narrow peaks significantly exceeding the standard range of signal amplitudes. A single peak may be caused by a random impact of a reflected particle into the nozzle. Repeated peaks and other distortions may be an indication of a sensor fault. In all cases, deviations from normal vibrations should be detected and excluded from the signal used for pressure estimation. Single or short-time deviations can simply be skipped. Long-time deviations and faults should be indicated to the equipment operator.

Practical investigation shows that energy entropy can be used to effectively detect needle peaks. In general, entropy is sensitive to very short and energy-rich deviations of a signal from the normal. In our algorithm used for abnormality detection, the signal was segmented into short segments of 500 samples, and each segment was further divided into *K* = 7 sub-segments. Then, the energies of the sub-segments were related to the energy of the entire segment. Finally, the energy entropy *H* in the *j*-th segment was computed as follows:

(5) $$H(j)=-\sum_{k=1}^{K}e_{k}(j)\ln\left[e_{k}(j)\right]$$

where *e_k_* are the relative energies. The full algorithm is described in [30].

**Figure 8:**
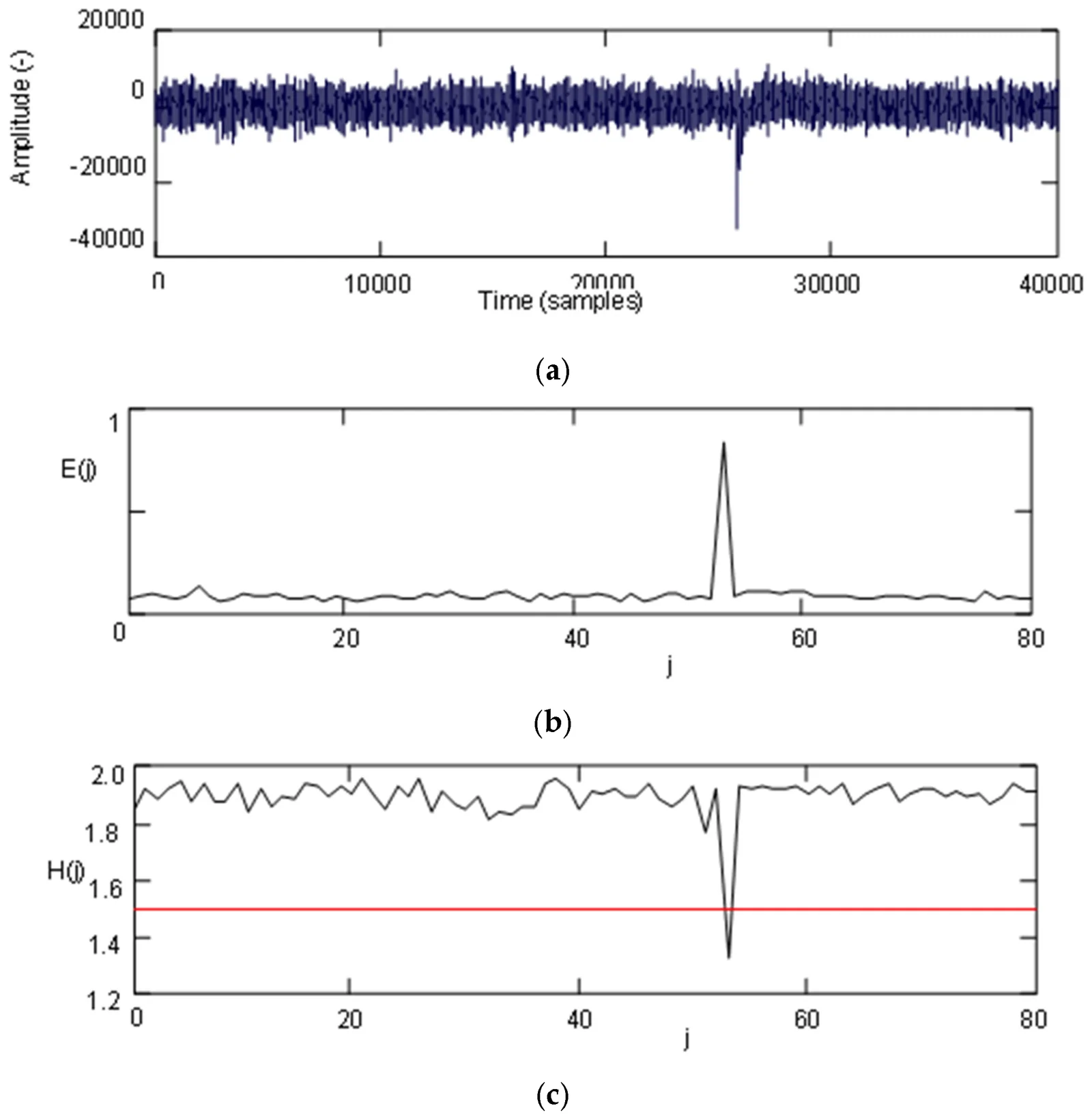
Vibration signal affected by a peak (**a**), corresponding short-time energy (**b**) and sequence of entropy values (**c**) with a threshold of 1.5 indicated for abnormality detection.

Figure 8 shows the entropy values for a standard vibration signal containing two spurious peaks. When a peak occurs, the entropy curve *H*(*j*) drops below the set threshold and thus, an abnormality is detected.

### 5.3. Exploring the Effective Implementation of Accelerometers

**Figure 9:**
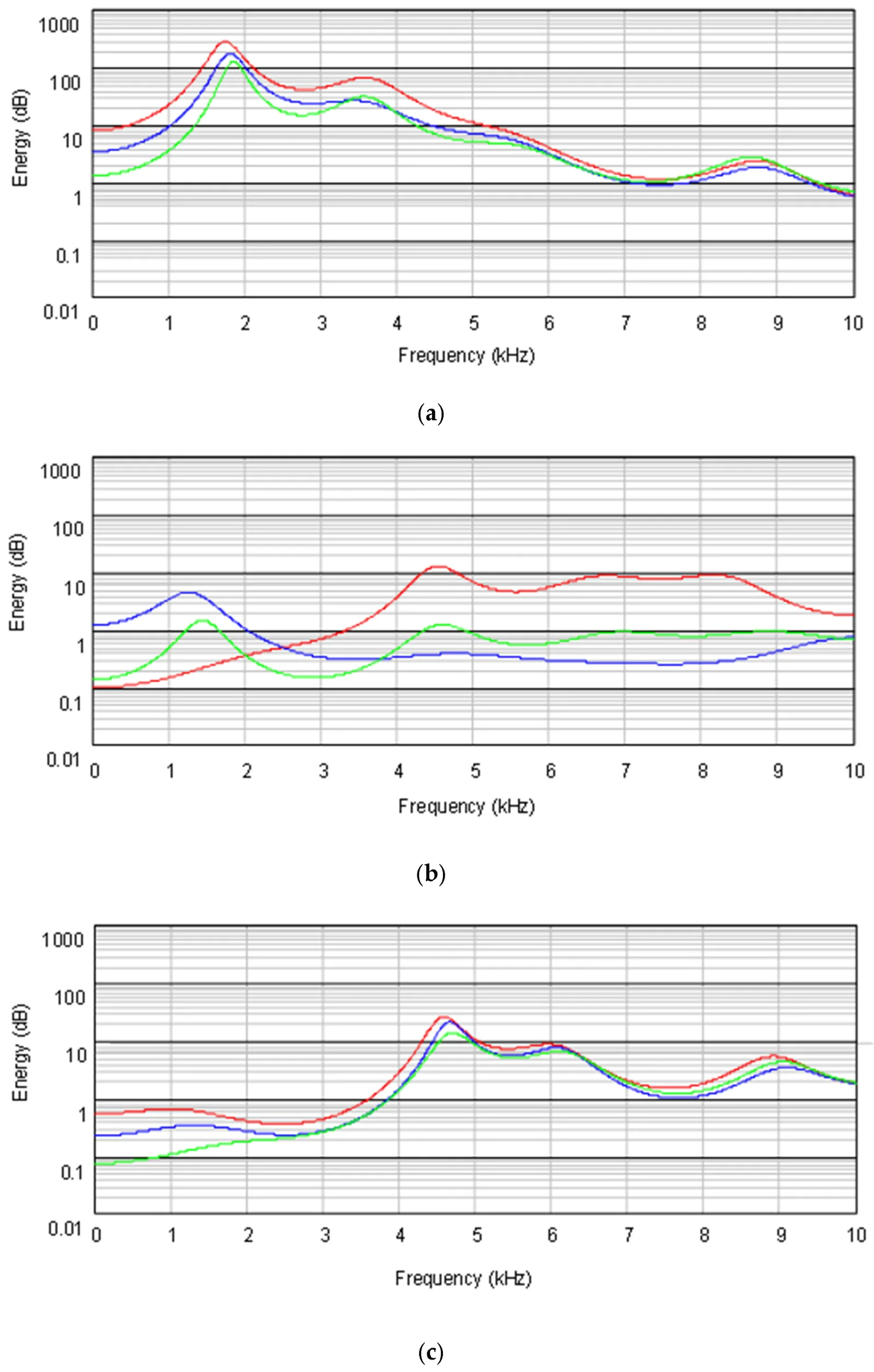
Spectra of the axial vibrations obtained from accelerometers A1 (**a**), A2 (**b**), and A3 (**c**) with color-coded pressure resolution: red line represents 3 bar, blue line 5 bar, and green line 7 bar.

All measurements presented in this section were performed for abrasive blasting at pressures of 3, 5, and 7 bar, 50% steel abrasive and a nozzle of 8 mm. First, the three types of potentially suitable accelerometers introduced in Section 3 were tested. The same blasting was measured simultaneously with two types of accelerometers, one sensor of each type located in the axial and one in the radial direction. The resulting vibration spectra in both observed directions showed a similar overall trend. The signals from A1 and A3 gave straightforward changes in the spectrum, with increasing pressure reflected by a decreasing value of the spectrum. Such a relationship did not apply to the signals from A2. Accelerometer A2 was therefore excluded from further measurements. Figure 9 illustrates typical comparisons of spectra obtained from axial vibrations. The spectra are plotted up to 10 kHz, but only the lower part up to 2000 Hz is relevant.

To capture vibrations optimally distinguishing the blasting pressure, the placement of A3 accelerometers in three different positions on the nozzle was tested as follows:

- upper position (on the nozzle holder);
- middle position (approximately in the middle of the nozzle);
- lower position (on the lower edge of the nozzle, i.e., closest to the end of the nozzle).

**Figure 10:**
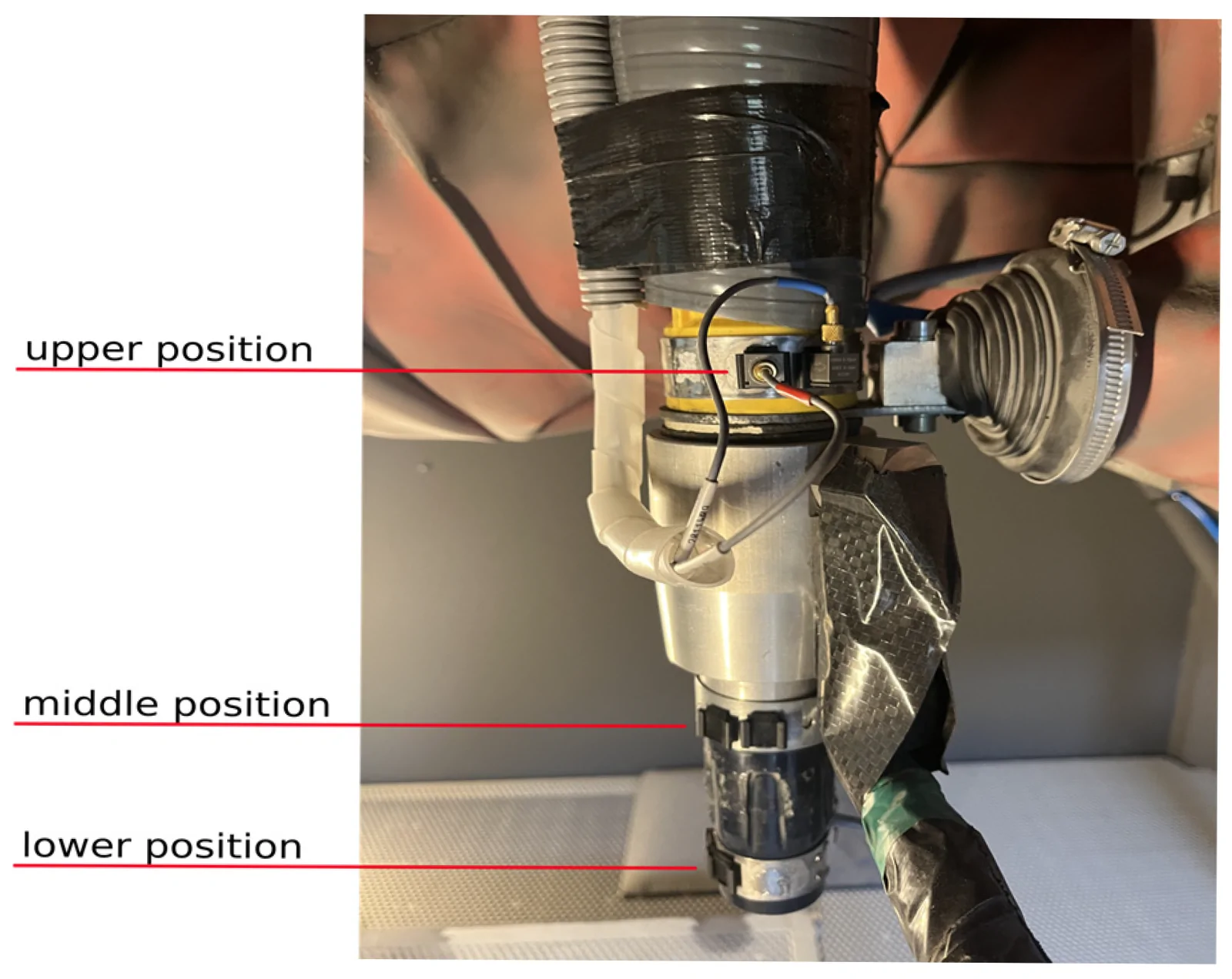
Photo of the outlet nozzle with marked positions for accelerometer placement.

**Figure 11:**
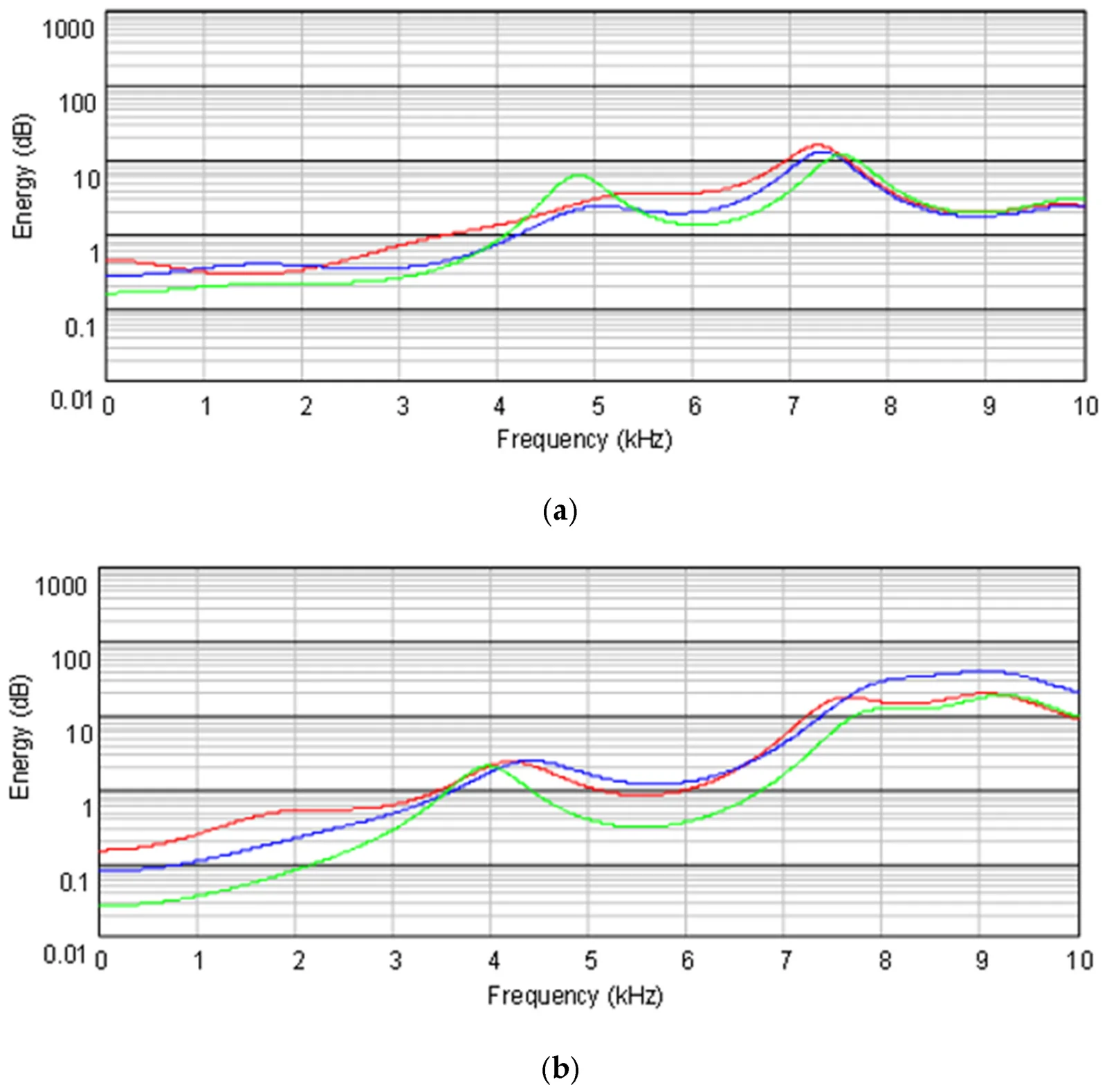
Spectra of the axial vibrations in the middle (**a**) and lower (**b**) positions.

The photo in Figure 10 shows the placement of two accelerometers when measuring in the upper position. Two pairs of black square clips can also be seen attached to metal rings in the middle and lower positions, ready for moving the accelerometers to these positions. In each position, measurements were made simultaneously in the radial and axial directions. Figure 11 shows typical vibration spectra in the middle and lower positions. For comparison, the spectra in the upper position are shown in Figure 9c.

According to the results, placing the accelerometers in the upper and lower positions appears to be better than in the middle position. When comparing the upper and lower positions, the spectral values in the relevant band up to 2000 Hz are slightly higher in the upper position. From an operational point of view, the significant difference is that the middle and lower positions are on the nozzle, while the upper position is on the nozzle holder. In this case, the accelerometer mounting can remain unchanged for all nozzles used. Since most blasting equipment has replaceable nozzles of various types and, moreover, it is necessary to replace worn nozzles with new ones, we focused in the further analysis only on the signals from the accelerometers in the upper position.

### 5.4. Classification of Vibration Signals

All practical tests based on LPC spectra were performed for blasting with steel abrasive and a nozzle with a diameter of 8 mm. First, the optimal frequency subband for resolving blasting pressures was investigated in each measurement scenario. The optimal subband in terms of center frequency and bandwidth was sought according to the following criterion:

(6) $$Q(\text{Low, High})=\frac{1}{\mathrm{High}-\mathrm{Low}}\sum_{f=\mathrm{Low}}^{\mathrm{High}}\frac{\mathrm{Total spectral difference}}{\mathrm{Average of spectra}}.$$

The total observed frequency range was limited from below by the minimum operating frequency of the accelerometers used and from above by the value of 2000 Hz, which is lower than the maximum operating frequency of the accelerometers. Higher *Q* values indicate a potentially better ability of the spectral band for pressure discrimination. A frequency search resulted in a subband of 0–1400 Hz for signals from A1 accelerometer and 0–400 Hz for signals from the A3 accelerometer. These subbands were used in the subsequent tests.

**Figure 12:**
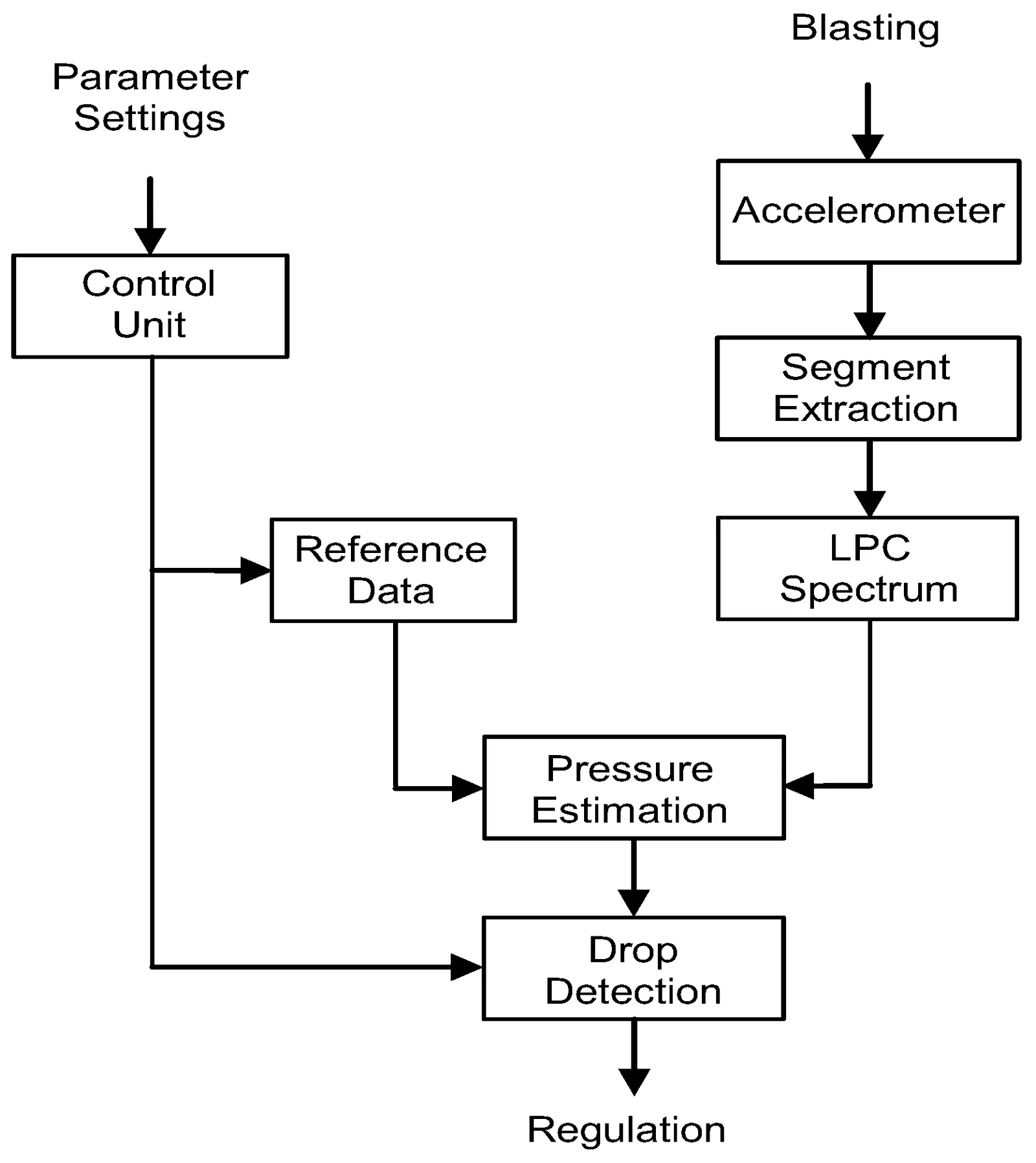
Block diagram of the proposed method.

Figure 12 illustrates the principle of the entire target method for determining the actual pressure during blasting for subsequent pressure adjustment. The tested 5-s segments are repeatedly extracted from the vibration signal, which is continuously sensed by the accelerometer. The LPC spectrum of each segment is then compared to the reference data using the squared Euclidean distance as a measure of similarity. The currently estimated pressure is related to the required, i.e., set pressure, and if a drop is detected, an automatic regulation process should be activated.

Multiple reference spectral data needed for comparison with the current spectrum are stored in memory and structured for all combinations of blasting pressure and abrasive ratio in the mixture. The selection of the appropriate reference data can be made automatically according to the blasting parameters that the operator must set before starting the blasting process. The values of the required pressure and abrasive ratio can be directly transferred from the blasting machine control unit to the “Reference Data” block. Similarly, the required pressure value can be transferred to the “Drop Detection” block in order to assess the current pressure drop.

This study deals only with the core of the developed method and focuses on the signal processing between the sensor and the “Pressure Estimation” block in Figure 12. A partial goal of the signal processing was the classification of vibration signals into classes according to blasting pressure. Due to the short and smooth supply of the blasting mixture from the compressor to the nozzle in the laboratory blasting machine used, the set blasting values were considered as the reference values. The LPC-spectrum can be directly used to classify vibration signals with a resolution of 1 bar and 2 bar. For a resolution of 0.5 bar, additional features derived from the spectrum need to be added. The best results were achieved in tests performed with signals generated at 50% abrasive in the mixture and pressures of 3, 5, and 7 bar, i.e., with a resolution of 2 bar. The detailed results are summarized in Table 2 and Table 3 in the form of confusion matrices for each accelerometer, with axial and radial vibrations being processed separately. The results summarized in the confusion matrices show that the middle pressure of 5 bar was classified with the worst efficiency due to confusion with pressures of 3 and 7 bar. On the other hand, pressure of 7 bar was never classified as a pressure of 3 bar.

To evaluate the overall success rate, three standard metrics were used: recall, precision and accuracy. These metrics are mathematically expressed as the following ratios:

(7) $$\mathrm{Recall}=\frac{\mathrm{TP}}{\mathrm{TP}+\mathrm{FN}}\cdot 100$$

(8) $$\mathrm{Precision}=\frac{\mathrm{TP}}{\mathrm{TP}+\mathrm{FP}}\cdot 100$$

(9) $$\mathrm{Accuracy}=\frac{\mathrm{TP}}{\mathrm{All tested signals}}\cdot 100$$

where *TP* is true positives, *TN* is true negatives, *FP* is false positives, and *FN* is false negatives. High values of *Recall*, *Precision*, and *Accuracy* indicate better functionality of the evaluated method.

The results in Table 2 and Table 3 (confusion matrices) are expressed in terms of recall, precision, and accuracy in Table 4 and Table 5. The best accuracy of 90.8% was achieved with the A1 accelerometer for axial vibrations. A significant decrease in accuracy to 74.0% occurred in the axial vibration classification with 100% abrasive in the mixture. It should be noted that such an abrasive ratio is not widely used in practice. The results for 100% abrasive ratio are shown in Table 6. In general, lower classification accuracy was achieved with a finer resolution of 1 bar for vibrations with pressures from 3 to 7 bar. For comparison, the results for 50% abrasive ratio are illustrated in Table 7. The *Recall* and *Precision* values in Table 4, Table 5, Table 6 and Table 7 are the averages of the individual pressure evaluations in the tested classification.

The results in Table 4, Table 5 and Table 7 (i.e., for 50% abrasive ratio) are based on signal processing from 5 recordings with a pressure of 3 bar and 4 recordings with each of the other pressures. Each reference spectrum for individual pressures was created as the average of 10 spectra obtained from 5-s segments of a single recording in the training phase. The remaining recordings (i.e., four signals at 3 bar and three signals at each of the other pressures) were used for testing. Each pressure was tested with a group of 41 to 67 signal segments. The results in Table 6 (i.e., for 100% abrasive ratio) are based on signal processing from three recordings at each pressure. In this case, the reference spectra for each pressure were created as the average of seven spectra obtained from 5-s segments of one recording. The remaining two recordings were used for testing. Each pressure was tested with a group of 26 to 31 signal segments. All signal segments used to create reference spectra, as well as signal segments used for testing, were free of abnormalities according to the check described in Section 5.2.

## 6. Conclusions

This paper is the first report on a new method leading to the estimation of actual blasting pressure. The following conclusions can be drawn from the current results obtained:

1. Vibration signals at the nozzle are a promising source for further research into non-invasive estimation of the actual pressure in the blasting mixture;
2. Accelerometers of type 4534 and 4507 are applicable for vibration measurement.
3. The most suitable position of the accelerometers is on the nozzle holder;
4. Five seconds is a sufficient length of vibration signals to obtain a stationary spectrum. Since the blasting process in practice usually takes tens of minutes and sometimes several hours, repeated signal acquisition for 5 s can be considered fast enough;
5. The LPC spectrum of nozzle vibrations at low frequencies up to 1400 Hz can be used as an indicator of blasting pressure;
6. In the 2-bar resolution with 50% abrasive ratio, the classification accuracy ranged between 80% and 90%. The best accuracy of 90.8% was achieved for axial vibration;
7. In the 1-bar resolution with 50% abrasive ratio, the classification accuracy decreased to 60% and should be improved with further research.

This research, initiated out of the need to solve a practical situation, will continue. First, it will be necessary to expand and complete the database of vibration signals. In future work, we will try to test the effectiveness of other features based on the spectrum used so far. For example, we plan to look for a suitable combination of mel-frequency cepstral coefficients [31], particularly, a variant with a Gaussian filter bank [32] that can faithfully model human noise perception. In general, other suitable features for processing vibration signals can also be sought in the field of infrasound processing [33]. It might also be beneficial to try to involve artificial intelligence in signal processing.

## Figures and Tables

**Table 1:** Basic parameters of accelerometers used.

Parameter	Accelerometer Type		
	A1 Brüel&Kjaer 4534-B-001	A2 Brüel&Kjaer 4517	A3 Brüel&Kjaer 4507-B-004
Voltage sensitivity	98 mV/g ± 5%	10 mV/g ± 10%	98 mV/g ± 5%
Measuring range	±700 g	±500 g	±70 g
Mounted resonance frequency	38 kHz	75 kHz	18 kHz
Residual broadband noise	0.5 mg	6 mg	< 0.35 mg
Frequency range (±10% in amplitude response)	0.2 Hz–12.8 kHz	1 Hz–20 kHz	0.3 Hz–6 kHz (0.3 Hz–1.5 kHz with dry- mounted clip)
Design	Shear	Shear	ThetaShear
Dimensions	14.8 mm × 14.8 mm × 13.5 mm	6.35 mm × 8.15 mm × 3.8 mm	10 mm × 10 mm × 10 mm
Weight	8.6 g	0.6 g	4.6 g
Mounting	10–32 UNF threaded	Adhesive	Mounting clip

**Table 2:** Confusion matrices in percent for axial and radial vibrations from A1 accelerometer.

Pressure		Labeled					
		Axial Vibration			Radial Vibration		
**True**		3 bar	5 bar	7 bar	3 bar	5 bar	7 bar
	3 bar	93.9	3.0	3.0	90.9	6.1	3.0
	5 bar	9.4	84.4	6.2	30.0	60.0	10.0
	7 bar	0	4.5	95.5	0	4.2	95.8

**Table 3:** Confusion matrices in percent for axial and radial vibrations from A3 accelerometer.

Pressure		Labeled					
		Axial Vibration			Radial Vibration		
**True**		3 bar	5 bar	7 bar	3 bar	5 bar	7 bar
	3 bar	96.4	3.6	0	96.9	3.1	0
	5 bar	17.4	69.5	13.1	25.0	65.0	10.0
	7 bar	0	8.7	91.3	0	15.0	85.0

**Table 4:** Evaluation of vibration classification with 2-bar resolution and 50% abrasive ratio using A1 accelerometer during blasting.

Vibration	Recall (%)	Precision (%)	Accuracy (%)
Axial	91.3	90.6	90.8
Radial	82.2	82.6	81.5

**Table 5:** Evaluation of vibration classification with 2-bar resolution and 50% abrasive ratio using A3 accelerometer during blasting.

Vibration	Recall (%)	Precision (%)	Accuracy (%)
Axial	85.8	86.3	86.6
Radial	82.3	84.1	84.9

**Table 6:** Evaluation of vibration classification with 2-bar resolution and 100% abrasive ratio using A1 accelerometer during blasting.

Vibration	Recall (%)	Precision (%)	Accuracy (%)
Axial	73.9	78.3	74.0
Radial	80.9	84.1	81.1

**Table 7:** Evaluation of vibration classification with 1-bar resolution and 50% abrasive ratio using A1 accelerometer during blasting.

Vibration	Recall (%)	Precision (%)	Accuracy (%)
Axial	58.5	63.3	60.2
Radial	65.3	68.1	65.7

## Data Availability

The raw data supporting the conclusions of this article will be made available by the authors on request.

## Appendix A

For a periodic or stationary random signal, the computation of prediction coefficients is usually performed for a short segment of the signal. There are three basic approaches to compute prediction coefficients: the autocorrelation method, covariance method, and lattice method. We used the autocorrelation method, which is almost exclusively used in audio signal processing due to its computational efficiency and inherent stability. This approach is described below.

The entire algorithm can be divided into two steps: (1) computation of the autocorrelation coefficients *R*(*k*) and then, (2) computation of the LPC coefficients *a_m_*. For a given discrete signal *s*(*n*), the short-time autocorrelation function is defined as follows:

(A1) $$R\left(k\right)=\sum_{n=1}^{N-k}s\left(n\right)s\left(n+k\right)$$

where *N* is the number of samples (i.e., the length) of the processed segment and the shift *k* (expressed in samples) plays the role of a variable parameter determining the autocorrelation coefficients *R*(*k*). The autocorrelation function is an even function, i.e., *R*(*k*) = *R*(*−k*).

When estimating signal samples using linear prediction, the error *e*(*n*) of the *n*-th sample is the difference between the original value *s*(*n*) and the predicted value $\hat{s}\left(n\right)$, expressed as follows:

(A2) $$e\left(n\right)\;=\;s\left(n\right)-\hat{s}\left(n\right)\;=\;s\left(n\right)-\sum_{m=1}^{M}a_{m}s\left(n-m\right).$$

Recalling that the linear prediction is defined in Section 4.3 (see Equation (1)), the computation of *a_m_* coefficients is based upon the requirement that the error sequence in finite time be minimal. The total error over the entire signal segment of length *N* is given as the sum of squared prediction errors, as follows:

(A3) $$E=\sum_{n=1}^{N}e^{2}\left(n\right)=\sum_{n=1}^{N}{\left[s\left(n\right)-\sum_{m=1}^{M}a_{m}s\left(n-m\right)\right]}^{2}.$$

The solution for the optimal coefficients *a_m_* is based on partial derivatives $\partial E/\partial a_{m}=0$ as follows:

(A4a) $$\begin{array}{c} \frac{\partial }{\partial a_{\mu }}\left\{\sum_{n=1}^{N}{\left[s\left(n\right)-\sum_{m=1}^{M}a_{m}s\left(n-m\right)\right]}^{2}\right\}=0\mu =1\ldots M\\ 2\sum_{n}\left[s\left(n\right)-\sum_{m=1}^{M}a_{m}s\left(n-m\right)\right]\left[-s\left(n-\mu \right)\right]=0 \end{array}$$

(A4b) $$\left[\sum_{n}s\left(n\right)-\sum_{n}\sum_{m}a_{m}s\left(n-m\right)\right]\left[-s\left(n-\mu \right)\right]=0$$

(A4c) $$\sum_{m}a_{m}\sum_{n}s\left(n-m\right)s\left(n-\mu \right)=\sum_{n}s\left(n\right)s\left(n-\mu \right)$$

(A4d) $$\sum_{m}a_{m}\sum_{n}s\left(n-m\right)s\left(n-m+m-\mu \right)=R\left(\mu \right)$$

(A4e) $$\sum_{m}a_{m}R\left(\left|m-\mu \right|\right)=R\left(\mu \right)$$

Note that the term *s*(*n − μ*) was extended to the form of *s*(*n – m + m − μ*), which does not change its value, but the equation can be written more compactly using the autocorrelation function *R*(|*m − μ|*).

The Equation (A4) can be expressed in matrix form as follows:

(A5) $$\left(\begin{array}{ccccc} R\left(0\right) & R\left(1\right) & R\left(2\right) & \ldots & R\left(M-1\right)\\ R\left(1\right) & R\left(0\right) & R\left(1\right) & \ldots & R\left(M-2\right)\\ . & . & . & \ldots & .\\ . & . & . & \ldots & .\\ R\left(M-1\right) & R\left(M-2\right) & R\left(M-3\right) & \ldots & R\left(0\right) \end{array}\right)\left(\begin{array}{c} a_{1}\\ a_{2}\\ .\\ .\\ a_{M} \end{array}\right)=\left(\begin{array}{c} R\left(1\right)\\ R\left(2\right)\\ .\\ .\\ R\left(M\right) \end{array}\right)$$

where *R*(0) to *R*(*M*) are the autocorrelation coefficients computed on the processed segment. Due to the special properties of the *M* × *M* matrix of autocorrelation coefficients, i.e., it is symmetric and has the same elements along the diagonal, the set of *M* linear equations can be solved by efficient algorithms.
